# Learning health systems, embedded research, and data standards—recommendations for healthcare system leaders

**DOI:** 10.1093/jamiaopen/ooaa046

**Published:** 2020-10-12

**Authors:** Rachel L Richesson

**Affiliations:** Department of Learning Health Sciences, University of Michigan Medical School, Ann Arbor, Michigan, USA

**Keywords:** data standards, data governance, electronic health record systems, pragmatic clinical trials, embedded research, healthcare systems

## Abstract

Learning health systems that conduct embedded research require infrastructure for the seamless adoption of clinical interventions; this infrastructure should integrate with electronic health record (EHR) systems and enable the use of existing data. As purchasers of EHR systems, and as critical partners, sponsors, and consumers of embedded research, healthcare organizations should advocate for EHR system functionality and data standards that will increase the capacity for embedded research in clinical settings. As stakeholders and proponents for EHR data standards, healthcare leaders should support standards development and promote local adoption to support quality healthcare, continuous improvement, innovative data-driven interventions, and the generation of new knowledge. “Standards-enabled” health systems will be positioned to address emergent and critical research questions, including those related to coronavirus disease 2019 (COVID-19) and future public health threats. The role of a data standards officer or champion could enable health systems to realize this goal.

## LAY SUMMARY

The use of data standards in electronic health record (EHR) systems can enable the use of clinical data to improve patient care and to increase our understanding of which treatments work best in the real world. However, data standards can be complicated and costly to implement. Data standards officers could be employed by healthcare systems to promote the value of data standards and coordinate their use in EHRs, ultimately enabling “research-ready” EHRs and learning health systems.

## ELECTRONIC HEALTH RECORD SYSTEMS ENABLE EMBEDDED RESEARCH

High healthcare costs and inadequate knowledge about the real-world effectiveness for most healthcare services are spurring the need for embedded research to learn which treatments and services are most effective. Researchers at NYU Langone Health System recently reported their instantiation of a *learning health system*^1^ by conducting randomized embedded trials to measure the impact of existing, often routine, activities and taking appropriate action.^2^ They have identified ineffective activities (eg, post-discharge telephone calls, appointment reminder letters), saving millions of dollars that were redirected to activities with greater impact. In the current state, few health systems can recognize activities with greater impact than those that address the urgent and unanswered clinical questions related to coronavirus disease 2019 (COVID-19) treatment approaches, screening, diagnosis, prediction, prevention, and prioritization for personal protective equipment and ventilators. The research investigations to answer these questions must include a broad range of patients and providers in healthcare delivery settings and will not have the luxury of narrow eligibility criteria or extensive data collection. In other words, these investigations will require research designs that are truly embedded in healthcare systems.

*Embedded research* is pragmatic by design, testing healthcare interventions that can be smoothly integrated into clinical workflows (often using electronic health record [EHR] systems), and collecting outcomes through usual care with little or no additional data collection.^3^ The authors of the NYU exemplar attribute their success to the careful selection of questions that could be explored within existing workflows and with data already captured in the EHR.^2^ The experience of other organizations conducting embedded clinical trials also confirms that the availability of data from EHR systems is an essential factor.^4^

Despite the potential of EHRs to enable embedded research, their complexity and variability can hinder the rapid configuration and conduct of studies required to address clinical and health delivery questions as they emerge. Variation in data and functionality from one EHR system to another means that organizations cannot easily replicate research or quality improvement studies without costly and time-consuming modification of code to configure interventions (eg, using alerts, pre-configured orders, or custom displays) or query data for reporting and analysis. In the NIH Health Care Systems Research Collaboratory, for example, many multi-site pragmatic trials reported that programming for data queries, data quality metrics, automated alerts and dashboards to support the trial, and analytic code had to be created anew *for every health system* participating in the trial.^5^ Unfortunately, this inability to easily conduct embedded research in different sites, settings, and patient populations also prevents the accumulation of *collective knowledge* of real-world effectiveness of healthcare interventions. COVID-19 is shining a bright spotlight on this national incapacity for embedded and multi-site research.

The COVID-19 pandemic has ignited national interest in standardized data and an appreciation for biomedical research. The audacious public endorsement of hydroxychloroquine by our President without supporting evidence is a sobering illustration of the critical need for national capacity to conduct efficient *randomized* clinical trials with affected populations to generate results that are swift, trustworthy, relevant, and actionable. The customization of EHR systems and supporting data standards for embedded clinical trials is the next great challenge for the field of informatics.

Reeves et al^6^ recently reported the quick optimization of the EHR to support screening, decision support, and reporting for COVID-19 in a statewide health system, and multiple national and international collaborations have formed impressively fast in response to the pandemic. However, the current lack of EHR interoperability has hindered the mobilization of large multi-site studies. Academic medical centers with data already mapped to a common data model are more able to contribute to national reporting and research efforts, but smaller healthcare organizations without this data readiness require time and technical support to connect into national efforts. Multiple registries, observational studies, and clinical trials for COVID-19 have emerged, but single site or even regional studies might not be sufficiently powered to address the questions that we need to answer—highlighting the need for interoperable systems and data standards that can support multi-site investigations on large or national scale.

## STANDARDS SUPPORT USE OF EHR DATA FOR RESEARCH AND INNOVATION

If health systems used standard specifications for representing data collected in EHRs, then programs to query, report, and analyze clinical data could be reused within and across organizations. To date, only a handful of data types are coded using nationally recognized standards (eg, diagnoses using International Classication of Diseases (ICD) codes, procedures using Current Procedural Terminology (CPT) codes). The widespread and continued use of these code systems for clinical, epidemiologic, and health services research—despite their known limitations—demonstrates the value of standardized data. Standardization of other data types, such as symptoms, patient features, goals, services, and outcomes, could facilitate research within and across health systems. Data standards can empower any clinician with a good idea^7^^,^^8^ (eg, bilirubin chart, cardiac risk calculator, custom growth chart display, or patient-directed medication management tool) to create an “app” and integrate it into *any* EHR system. Such standards-enabled innovation will offer providers and patients choices for how to view and act upon health data.^9^ The US Office of the National Coordinator is promoting standards^10–12^ to accelerate the use of health data, but the relevance and impact of these standards will ultimately depend upon the engagement of health systems whose providers and researchers will use them.

## RECOMMENDATIONS FOR HEALTH SYSTEMS TO EMBRACE STANDARDS AND ADVANCE THEIR MISSIONS

Using finite resources, health systems have an imperative to deliver the best care possible and need to continuously innovate, evaluate, and prioritize the services and apps they provide. As purchasers and custodians of EHR systems, healthcare system leaders can be engaged consumers who recognize the central role of data standards in a dynamic health and research ecosystem. To catalyze this, organizations should:

*Identify a data standards officer or champion* to investigate, promote, and coordinate the use of standards and reduce unnecessary variation in data collected across the health system. For example, lab tests could be natively coded in Logical Observation Identifiers Names and Codes (LOINC)^13^ for rapid comparison and querying of results (eg, microbiology, glucose). Pain assessments could be standardized. (A recent study of 10 organizations revealed dozens of structured data elements for pain, including many variations on the classic 0–10 pain intensity scale.^14^) There may be multiple operational definitions (ie, queries) for specific conditions across research studies, quality measurement reports, and registries,^15^ making it difficult to retrieve and reuse queries to identify patients with diabetes or other conditions (eg, chronic obstructive pulmonary disease, substance abuse, chronic pain, terminal illness, pregnancy). Designated individuals with a broad organizational view can identify opportunities where data standards will create efficiencies and enable the reuse of analytic programs and executable applications such as clinical decision support. In parallel, they can ensure that the organization is able to participate in networked research and leverage external data sources (eg, community-level health metrics, environmental data, or the national death index).

*Incorporate data standards into data governance and develop policies around new data collection based upon the principle of using and promoting data standards*. Regular examination of the utility and burden of data collection can reduce clinician frustration with EHRs and documentation requirements.^16^ Governance structures can address the thoughtful selection of new data elements, and retirement of data collection that serves no clear purpose. Healthcare organizations should strive for a set of core data elements that support many purposes—including embedded research, population health management, clinical decision support, and quality measurement. Local researchers should be able to propose additional data elements to support research in specific disease or clinical domains, but a standards officer could act as a data sentry to ensure that those new elements are represented in a standardized format, promoting consistent question modeling and leveraging the formal semantics of controlled terminologies. Similarly, common approaches to collecting patient-reported outcomes can support organizational goals for quality and value-driven care across different disease populations and health services.

*Participate in national standards efforts*. The standards officer can champion the role of data standards in disseminating research and successful innovation for adoption by other organizations. A local standards officer can coordinate organizational activities with standards development organizations (SDOs), and ensure that their EHR systems, data, and applications address local needs *and* comply with standards implementation approaches that will complement national research strategy^12^, registries or research networks. Standards officers can participate in SDOs to request and organize the extension of existing standards as needed. There are many SDOs,^17^ and health systems and professional medical societies can coordinate their participation for stronger impact. For example, the standards officer can encourage or assign others to actively participate in standards working groups that align with their interests (eg, clinical decision support, laboratory standardization, patient reported outcomes (PROs), and coordinate the sharing of information and cooperation with standards delegates within or across organizations to better understand standards with which they are less familiar.

*Develop infrastructure to implement emerging standards, including education, coordination, and communication with other stakeholders*. Health systems leaders need to capitalize on the national paradigm shift of the US Food and Drug Administration toward active surveillance, real-world evidence, and embedded research—all of which require data standards. The emerging HL7 Fast Healthcare Interoperability Resources (FHIR) standard is a modern and extensible data exchange standard that has garnered interest from big industry like Apple, Google, Microsoft, and Amazon, and endorsement by CMS.^18^ The NIH has formally encouraged researchers to use FHIR to access EHR data and to share research data sets.^19^ The NLM is exploring and promoting FHIR to extract genomic and phenotype data from NIH databases and to integrate data from genetic test results into EHRs.^20^

Adoption of the FHIR specification, however, will not eliminate the need to coordinate and standardize data collection. For example, if one organization collects and codes smoking status one way (eg, Current, Former, Never) and another codes it differently (eg, Yes, No), then combining or comparing data between organizations will require transformation and be subject to variation or error. Similarly, using FHIR to evaluate an opioid treatment program will likewise be problematic if the intervention codes or pain assessment values vary across units or sites. Because FHIR is a new standard, there are ambiguities on how to integrate complex coding systems such as the Systematized Nomenclature of Medicine Clinical Terms (SNOMED CT). Furthermore, a proliferation of FHIR profiles with different specifications for coding systems or required elements can lead to confusion and duplication of effort if left unchecked. Organizations can use their standards officer to identify optimal and consistent approaches for representing data using FHIR, including composite measures computed from existing data and external data sources. Furthermore, organizations can help develop and inform methods to query and transmit *population level* data (ie, “batch FHIR”) that is needed for research and population health. Consistent approaches for integrating FHIR with other standards (eg, CDS Hooks) can facilitate the development use of EHR-integrated decision support to increase the adoption of treatments shown to be effective. (CDS Hooks^21^ identifies common actions [eg, open record, create care plan, order medication] that can be used to “hook” CDS alerts or reminders into the EHR and hence the clinical workflow.)

Undoubtedly, many healthcare organizations have implemented various data standards and are addressing the above recommendations through existing data governance structures. However, these organizations will benefit from a designated standards advocate role, occupied by a person who can bring the technical knowledge about various standards structures and personal connections with SDOs to advance a vision for a standards-enabled and research-ready organization. This person can ensure that when new data collection is proposed—eg, for mobile apps, questions about emerging risk factors for new syndromes or infectious diseases such as COVID-19— appropriate existing standards  are applied or a path to standardization that others can adopt is quickly identified.

The activities of a standards officer could take many forms and the role does not necessarily require a full-time effort or a single person. One or more individuals with some allocated time and dedicated perspective of standards implementation could be coordinated to maximize the efficiency and strategic use of standards to facilitate organizational goals and national collaborations. This proposed role of standards officer complements existing Chief Medical Information Officer or Chief Research Informatics Officer roles but is markedly different in its focus on the science and practice of data standards development, implementation, and evaluation. A data standards officer can bring deep technical knowledge of an array of standards for different data types and clinical disciplines, an understanding of dependencies and unintended consequences, and access to professional networks with varied experience implementing and using standards in different settings and programs. Leaders from the C-suite can certainly champion for standards, but their commitment to organizational operations and priorities leaves limited time for the detailed understanding of data standards needed for successful implementation. A standards officer’s focus on broader goals of system interoperability, sustainability, and scalability can facilitate not only the development of internal research infrastructure and learning health system capabilities, but also can foster *external* collaborations and interactions (eg, with other research or learning networks or other data sources), offering extended opportunities to deliver and assess high-value healthcare and ultimately achieve the Quadruple Aim.^16^

Despite the potential impacts that data standards officer might facilitate, in order to justify the costs, the value of this role must be tied to immediate benefits to the health system. The foundation for the “shovel ready” data and “analytic pipelines” to which modern health systems aspire is data that is collected using well-documented and consistent procedures and is represented by data elements and coding systems that are explicit and standardized. This standardization can enable the rapid aggregation and analysis of enterprise data through the reuse of query tools and analytic code. Furthermore, the widespread and consistent adoption of data representation and exchange standards can facilitate health systems of *any* size to participate in both observational and interventional research studies, ultimately enabling sufficient sample size and diversity to meaningfully impact population health.

The recommendations around standards presented here are not novel, but they have not been widely adopted due to socio-technical challenges long familiar to informaticians, including the tension between system designers and end-users around the burden of clinical documentation relative to the perceived utility of the data collected. The value propositions for data standards themselves have been a challenge for informatics, as standards-enabled data aggregation and exchange appear to impact the “public good” rather than those that purchase EHR systems or collect the data. For some data standards, such as ICD-10-CM, financial incentives for organizations are quite clear. The value propositions for more complex standards such as SNOMED CT or FHIR, or those relevant to only specific disease areas (eg, common data elements, PRO questions), are even more challenging to develop. The recommendations around data standards presented here are not tenable without the realignment of incentives that drive the behavior of health systems. However, the trajectory toward value-based care payment is established and gaining momentum, and as we progress toward that goal, health system leaders, stakeholders, and policymakers will increasingly expect and demand adequate data to measure and compare the value of different interventions and approaches to care. In the meanwhile, informatics professionals focused on standards and working with health systems can and should create the infrastructure for data and embedded clinical trials that is needed and expected, and will inevitably be demanded.

Embedded research will require near real-time outcomes and access to outcome data outside of visits. Standards support this and are a key component for “research readiness” of an organization. Healthcare systems are positioned to demand and adopt data representation and exchange standards that will facilitate healthcare innovation and subsequent evaluation using embedded research. Consensus and coordination will be required to ensure that the standards evolve to support the needs of learning health systems. Standards are not “one and done.” They are dynamic and will require user communities to define—and refine—how they should be applied in different contexts. New standards will always be needed, especially in areas of innovation (eg, new devices, patient-generated, and patient-reported data). These innovations and emergent health issues will create a continuous need for evaluation driven by data. Data standards will support these evaluations and are critical and dynamic component of learning health systems. Using one or more designated data standards officers, healthcare systems can and should actively participate in the ongoing development and promotion of data standards that will support quality healthcare delivery, continuous quality improvement, and the generation of new knowledge.

## FUNDING

This work was supported in part by the National Institutes of Health (NIH) Common Fund, through a cooperative agreement (U54 AT007748) from the Office of Strategic Coordination within the Office of the NIH Director. The opinions expressed in this paper do not represent the official views of the NIH.

## AUTHOR CONTRIBUTIONS

The sole author RLR developed the ideas in this manuscript, wrote and edited the text, and revised the manuscript.

## CONFLICT OF INTEREST STATEMENT

None declared.
